# Susceptibility of important Gram-negative pathogens to tigecycline and other antibiotics in Latin America between 2004 and 2010

**DOI:** 10.1186/1476-0711-11-29

**Published:** 2012-10-22

**Authors:** Liliana Fernández-Canigia, Michael J Dowzicky

**Affiliations:** 1 Laboratorio de Microbiología, Hospital Aleman, Pueyrredón 1640, PB, Ciudad Autónoma de Buenos Aires, Argentina; 2 Pfizer Inc, 500 Arcola Road; E-Dock, Collegeville, PA, 19426, USA

**Keywords:** Tigecycline, Latin America, Resistance, Susceptibility, Carbapenems

## Abstract

**Background:**

The Tigecycline Evaluation and Surveillance Trial (T.E.S.T.) is a global surveillance study of antimicrobial susceptibility. This study reports data from Gram-negative isolates collected from centers in Latin America between 2004 and 2010.

**Methods:**

Consecutive bacterial isolates were tested at each center using broth microdilution methodology as described by the Clinical Laboratory Standards Institute (CLSI). Susceptibility was determined using the CLSI interpretive criteria. For tigecycline the US Federal Drug Administration (FDA) criteria were used.

**Results:**

A total of 16 232 isolates were analyzed. Susceptibility to imipenem, meropenem, and tigecycline was >95% against both non-extended-spectrum β-lactamase (ESBL) and ESBL producing *Escherichia coli*. Susceptibility to amikacin was also >95% for non-ESBL *E. coli*. 24.3% of *E. coli* were ESBL producers, ranging from 11.2% (58/519) in Colombia to 40.3% (31/77) in Honduras. Greater than 90% of non-ESBL *Klebsiella pneumoniae* were susceptible to tigecycline, carbapenems and amikacin. 35.3% of *K. pneumoniae* were ESBL producers, ranging from 17.2% (36/209) in Venezuela to 73.3% (55/75) in Honduras, with only imipenem and tigecycline maintaining >90% susceptibility. Greater than 90% of *Klebsiella oxytoca*, *Enterobacter* spp., and *Serratia marcescens* were susceptible to amikacin, carbapenems and tigecycline. The highest rates of susceptibility against *Acinetobacter baumannii* were seen for minocycline (89.4%) and imipenem (62.5%), while 95.8% of the *A. baumannii* isolates displayed an MIC ≤2 μg/mL for tigecycline.

**Conclusions:**

In this study carbapenems and tigecycline remain active against Enterobacteriaceae and *A. baumannii*; however, there is cause for concern with carbapenem non-susceptible isolates reported in all countries included in this study.

## Background

Tigecycline is a glycylcycline licensed by the US Food and Drug Administration (FDA) for the treatment of complicated skin and skin structure infections (cSSSI), complicated intra-abdominal infections (cIAIs) and community acquired bacterial pneumonia (CAP). The Tigecycline Evaluation and Surveillance Trial (T.E.S.T.) is a global surveillance study with the aim of assessing and reporting the antimicrobial susceptibility of tigecycline and comparator agents globally, regionally, and for individual countries. T.E.S.T. was initiated in 2004 and to date 60 countries have contributed with Gram-positive and Gram-negative isolates and susceptibility data. Antimicrobial surveillance studies, such as T.E.S.T., play a key role in charting antimicrobial resistance.

The Latin American region is recognized as facing a significant challenge with high levels of antimicrobial resistance among important Gram-negative organisms including *Escherichia coli* and *Klebsiella* spp. and the non-fermenters *Acinetobacter* spp. and *Pseudomonas aeruginosa*[1-3]. In recent years, extended-spectrum β-lactamases (ESBLs) have increased in type and frequency among Enterobacteriaceae and carbapenemases have emerged [4,5]. In the case of the non-fermentative Gram-negative bacilli multidrug-resistance is an increasing problem with limited, or no treatment option [6].

In this report we present data from the Latin American region of Gram-negative isolates collected between 2004 and 2010. The isolates collected between 2004 and 2007 were previously reported by Rossi et al. [7].

## Methods

### Organism collection

Gram-negative isolates were collected from 12 countries in Latin America between 2004 and 2010. Centers were distributed as follows: 12 in Argentina, 3 in Brazil, 5 in Chile, 14 in Colombia, 1 in El Salvador, 4 in Guatemala, 2 in Honduras, 1 in Jamaica, 15 in Mexico, 1 in Nicaragua, 2 in Panama, and 6 in Venezuela. The Gram-negative isolates submitted were consecutive and determined to be clinically significant using local criteria. Permissible clinical sources included blood, respiratory tract, urine (limited to no more than 25% of all isolates), skin, wound, and fluids. For each year, each participant center was required to identify and conduct susceptibility tests on *Acinetobacter* spp. (15), *E. coli* (25), *Enterobacter* spp. (25), *Serratia* spp. (10), *Klebsiella* spp. (25) and *Haemophylus influenzae* (15). A single isolate per patient was accepted. Inclusion in the study was independent of the patient’s medical history, previous antimicrobial use, sex and age. No banked or stored isolates were accepted.

### Antimicrobial susceptibility testing

Each study center carried out antimicrobial susceptibility testing using broth microdilution methodology (Sensititre® plates [TREK Diagnostic Systems, West Sussex, England] or MicroScan® panels [Siemens, Sacramento, CA, USA]) as described by the Clinical and Laboratory Standards Institute (CLSI) [8]. Gram-negative isolates were tested against amikacin, amoxicillin-clavulanate, ampicillin, cefepime, ceftazidime, ceftriaxone, imipenem, levofloxacin, meropenem, minocycline, piperacillin-tazobactam, and tigecycline. In 2006, unreliability of the imipenem testing led to a switch from MicroScan® panels with imipenem to Sensititre® plates with meropenem. The presence or abscence of β-lactamase among *H. influenzae* was determined using the preferred method of each center.

Quality control strains used in the testing were *E. coli* ATCC 25922 and *P. aeruginosa* ATCC 27853. Confirmation of isolate identification and management of a centralized database were performed by a central laboratory (Laboratories International for Microbiology Studies, a division of International Health Management Associates, Inc. [IHMA, Schaumburg, IL, USA]).

Antimicrobial susceptibility was determined using CLSI interpretive criteria [9,10]. For tigecycline, the FDA approved breakpoints, as provided in the package insert, were used [11].

### Extended spectrum β-lactamase (ESBL) determination

Testing for ESBL production was carried out on isolates of *E. coli* and *Klebsiella* spp. according to the CLSI guidelines [9]. The methodology used Mueller-Hinton agar (Remel, Inc., Lenexa, KS, USA) and cefotaxime (30 μg), cefotaxime-clavulanic acid (30/10 μg), ceftazidime (30 μg), and ceftazidime-clavulanic acid (30/10 μg) discs (Oxoid, Inc., Ogdensburg, NY, USA). Quality control was carried out using *K. pneumoniae* ATCC 700603 (ESBL-positive) and *E. coli* ATCC 25922 (ESBL-negative).

### Multidrug-resistant *Acinetobacter baumannii*

Multidrug resistance among isolates of *A. baumannii* was defined as resistance to levofloxacin, amikacin, carbapenems (imipenem and/or meropenem), ceftazidime and piperacillin-tazobactam.

## Results

**Figure 1:**
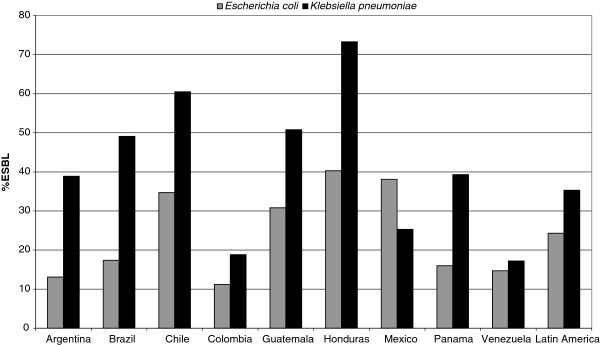
**Percentage of *Escherichia coli *and *Klebsiella pneumoniae *isolates identified as ESBL producers in each Latin American country**^**a **^**involved in T.E.S.T. (2004–2010). ***E. coli* N values: Argentina, 101/769; Brazil, 43/247; Chile, 94/271; Colombia, 58/519; Guatemala, 81/263; Honduras, 31/77; Mexico, 398/1044; Panama, 16/100; Venezuela, 32/218; Latin America, 870/3581. *K. pneumoniae* N values: Argentina, 270/694; Brazil, 105/214; Chile, 147/243; Colombia, 81/432; Guatemala, 96/189; Honduras, 55/75; Mexico, 191/754; Panama, 35/89; Venezuela, 36/209; Latin America, 1045/2962. ^a^ Data from El Salvador, Jamaica and Nicaragua are not included in the analysis by country because fewer than 50 isolates were collected; however, their data are included in the total for Latin America.

Antimicrobial susceptibility data on 16 232 Gram-negative isolates collected in Latin America between 2004 and 2010 are presented in Table 1. Susceptibility among the *E. coli* isolates (both ESBL and non-ESBL producers) was >95% for carbapenems and tigecycline. Susceptibility to amikacin was also >95% against non-ESBL producing *E. coli* (MIC_90_ 8 μg/mL) but decreased to 89.7% against ESBL producers (MIC_90_ 32 μg/mL). A total of 24.3% of the *E. coli* collected from Latin America were identified as ESBL producers with percentages of ESBL production varying from 11.2% (58/519) in Colombia to 40.3% (31/77) in Honduras (Figure 1). Data on susceptibility to imipenem and meropenem by country are presented in Table 2. Among *E. coli* isolates, ESBL producers displayed slightly lower susceptibility to meropenem than non-ESBL producing isolates.

**Table 1:** Antimicrobial activity against Gram-negative organisms collected from Latin America (2004 – 2010)

**Organisms/antimicrobial**		**MIC (mg/L)**			**Percentage**		
	**N**	**50**	**90**	**Range**	**S**	**I**	**R**
**non-ESBL *E. coli***							
Amikacin	2711	2	8	≤0.5 to ≥128	97.2	1.2	1.7
Amoxi/clav	2711	8	32	≤0.12 to ≥64	60.5	21.2	18.4
Ampicillin	2711	≥64	≥64	≤0.5 to ≥64	28.6	1.5	69.9
Cefepime	2711	≤0.5	4	≤0.5 to ≥64	94.3	2.1	3.6
Ceftazidime^a^	2711	≤8	16	≤1 to ≥64	-	-	11.7
Ceftriaxone	2711	≤0.06	32	≤0.06 to ≥128	82.0	2.3	15.6
Imipenem	485	0.25	0.5	≤0.06 to ≥32	98.6	0.6	0.8
Levofloxacin	2711	0.25	≥16	≤0.008 to ≥16	60.9	3.0	36.2
Meropenem	2226	≤0.06	0.12	≤0.06 to ≥32	98.6	0.4	1.0
Minocycline	2711	4	16	≤0.5 to ≥32	62.4	14.3	23.2
Pip/taz	2711	2	32	≤0.06 to ≥256	88.8	5.2	6.0
Tigecycline	2711	0.25	0.5	≤0.008 to ≥32	99.7	0.2	<0.1^c^
**ESBL *E. coli***							
Amikacin	870	4	32	≤0.5 to ≥128	89.7	5.1	5.3
Amoxi/clav	870	16	32	0.25 to ≥64	21.1	42.5	36.3
Cefepime	870	32	≥64	≤0.5 to ≥64	28.2	14.3	57.6
Ceftazidime^a^	870	16	≥64	≤1 to ≥64	-	-	65.5
Ceftriaxone	870	≥128	≥128	≤0.06 to ≥128	1.1	2.0	96.9
Imipenem	143	0.25	0.5	≤0.06 to 8	97.9	0.7	1.4
Levofloxacin	870	≥16	≥16	0.015 to ≥16	11.5	3.4	85.1
Meropenem	727	≤0.06	0.12	≤0.06 to ≥32	96.4	1.2	2.3
Minocycline	870	4	≥32	≤0.5 to ≥32	52.3	14.6	33.1
Pip/taz	870	8	64	≤0.06 to ≥256	73.9	16.1	10.0
Tigecycline	870	0.25	0.5	≤0.008 to 4	99.8	0.2	0.0
**Non-ESBL *K. pneumoniae***							
Amikacin	1917	2	8	≤0.5 to ≥128	93.4	1.8	4.8
Amoxi/clav	1917	4	≥64	0.25 to ≥64	67.5	10.0	22.5
Cefepime	1917	≤0.5	16	≤0.5 to ≥64	87.7	2.8	9.4
Ceftazidime^a^	1917	≤8	32	≤1 to ≥64	-	-	17.2
Ceftriaxone	1917	≤0.06	≥128	≤0.06 to ≥128	77.1	1.4	21.5
Imipenem	275	0.5	0.5	≤0.06 to ≥32	98.9	0.0	1.1
Levofloxacin	1917	0.06	≥16	≤0.008 to ≥16	80.1	2.1	17.7
Meropenem	1642	≤0.06	0.25	≤0.06 to ≥32	94.6	1.0	4.4
Minocycline	1917	4	≥32	≤0.5 to ≥32	65.8	11.1	23.1
Pip/taz	1917	4	≥256	≤0.06 to ≥256	79.6	6.2	14.2
Tigecycline	1917	0.5	1	≤0.008 to ≥32	96.9	2.3	0.8
**ESBL *K. pneumoniae***							
Amikacin	1045	8	≥128	≤0.5 to ≥128	71.2	8.3	20.5
Amoxi/clav	1045	32	≥64	≤0.12 to ≥64	13.1	30.6	56.3
Cefepime	1045	32	≥64	≤0.5 to ≥64	29.2	12.1	58.8
Ceftazidime^a^	1045	32	≥64	≤2 to ≥64	-	-	81.1
Ceftriaxone	1045	≥128	≥128	≤0.06 to ≥128	1.0	1.2	97.8
Imipenem	199	0.5	1	≤0.06 to 16	96.0	2.5	1.5
Levofloxacin	1045	8	≥16	≤0.008 to ≥16	38.2	5.5	56.4
Meropenem	846	≤0.06	2	≤0.06 to ≥32	89.0	2.4	8.6
Minocycline	1045	8	≥32	≤0.5 to ≥32	49.0	17.1	33.9
Pip/taz	1045	64	≥256	0.12 to ≥256	34.5	20.8	44.7
Tigecycline	1045	0.5	2	0.03 to 16	93.7	4.9	1.4
***K. oxytoca***							
Amikacin	311	2	8	≤0.5 to ≥128	94.9	1.6	3.5
Amoxi/clav	311	4	32	0.25 to ≥64	69.1	11.9	19.0
Cefepime	311	≤0.5	16	≤0.5 to ≥64	85.5	5.8	8.7
Ceftazidime^a^	311	≤8	32	≤1 to ≥64	-	-	20.9
Ceftriaxone	311	0.12	≥128	≤0.06 to ≥128	68.5	2.9	28.6
Imipenem	76	0.5	0.5	≤0.06 to 1	100	0.0	0.0
Levofloxacin	311	0.06	≥16	≤0.008 to ≥16	81.0	1.3	17.7
Meropenem	235	≤0.06	0.12	≤0.06 to 16	97.4	1.3	1.3
Minocycline	311	2	16	≤0.5 to ≥32	77.5	10.3	12.2
Pip/taz	311	2	128	≤0.06 to ≥256	83.6	6.1	10.3
Tigecycline	311	0.25	1	0.06 to 4	97.7	2.3	0.0
***Enterobacter *spp.**							
Amikacin	2804	2	32	≤0.5 to ≥128	89.2	4.4	6.5
Amoxi/clav	2804	≥64	≥64	≤0.12 to ≥64	4.7	3.0	92.3
Cefepime	2804	≤0.5	≥64	≤0.5 to ≥64	81.4	4.6	14.1
Ceftazidime^a^	2804	≤8	≥64	≤1 to ≥64	-	-	40.5
Ceftriaxone	2804	1	≥128	≤0.06 to ≥128	51.9	2.6	45.5
Imipenem	493	0.5	1	≤0.06 to ≥32	95.9	2.6	1.4
Levofloxacin	2804	0.12	≥16	≤0.008 to ≥16	78.2	3.1	18.8
Meropenem	2311	≤0.06	0.5	≤0.06 to ≥32	94.3	1.9	3.8
Minocycline	2804	4	≥32	≤0.5 to ≥32	61.9	17.7	20.3
Pip/taz	2804	4	≥256	≤0.06 to ≥256	70.1	11.7	18.1
Tigecycline	2804	0.5	2	≤0.008 to ≥32	96.0	3.5	0.5
***S. marcescens***							
Amikacin	1126	2	64	≤0.5 to ≥128	82.6	7.2	10.2
Amoxi/clav	1126	≥64	≥64	≤0.12 to ≥64	4.4	2.9	92.6
Cefepime	1126	≤0.5	32	≤0.5 to ≥64	83.6	3.7	12.7
Ceftazidime^a^	1126	≤8	32	≤1 to ≥64	-	-	17.5
Ceftriaxone	1126	0.5	≥128	≤0.06 to ≥128	67.8	3.4	28.9
Imipenem	229	0.5	1	≤0.06 to 8	91.7	6.1	2.2
Levofloxacin	1126	0.25	4	≤0.008 to ≥16	86.2	4.4	9.3
Meropenem	897	≤0.06	0.25	≤0.06 to 8	96.2	1.8	2.0
Minocycline	1126	4	16	≤0.5 to ≥32	61.3	23.6	15.1
Pip/taz	1126	2	64	≤0.06 to ≥256	84.0	6.9	9.1
Tigecycline	1126	1	2	≤0.008 to 16	95.5	3.7	0.8
***H. influenzae***							
Amoxi/clav	908	0.5	1	≤0.12 to 16	99.3	0.0	0.7
Ampicillin	908	≤0.5	16	≤0.5 to ≥64	78.7	2.5	18.7
Cefepime	908	≤0.5	≤0.5	≤0.5 to 8	99.6	--	--
Ceftazidime^b^	902	≤8	≤8	≤8 to 16	--	--	--
Ceftriaxone	908	≤0.06	≤0.06	≤0.06 to 2	100	--	--
Imipenem	217	0.5	1	≤0.06 to 4	100	--	--
Levofloxacin	908	0.015	0.03	≤0.008 to 2	100	--	--
Meropenem	691	≤0.06	0.12	≤0.06 to 0.5	100	--	--
Minocycline	908	≤0.5	1	≤0.5 to 16	98.7	0.8	0.6
Pip/taz	908	≤0.06	≤0.06	≤0.06 to 4	99.7	0.0	0.3
Tigecycline	908	0.12	0.25	≤0.008 to 0.5	98.8	--	--
***A. baumannii***							
Amikacin	1806	64	≥128	≤0.5 to ≥128	30.4	12.0	57.6
Cefepime	1806	32	≥64	≤0.5 to ≥64	25.3	14.4	60.3
Ceftazidime	1806	≥64	≥64	≤1 to ≥64	18.5	7.8	73.8
Ceftriaxone	1806	≥128	≥128	≤0.06 to ≥128	10.5	11.1	78.4
Imipenem	307	2	≥32	≤0.06 to ≥32	62.5	3.9	33.6
Levofloxacin	1806	8	≥16	≤0.008 to ≥16	20.9	11.4	67.8
Meropenem	1499	≥32	≥32	≤0.06 to ≥32	33.9	5.5	60.6
Minocycline	1806	≤0.5	8	≤0.5 to ≥32	89.4	4.6	6.0
Pip/taz	1806	≥256	≥256	≤0.06 to ≥256	18.7	9.1	72.2
Tigecycline	1806	0.5	2	≤0.008 to ≥32	--	--	--
***P. aeruginosa***							
Amikacin	2734	4	≥128	≤0.5 to ≥128	71.8	8.1	20.0
Cefepime	2734	8	≥64	≤0.5 to ≥64	59.8	15.2	25.1
Ceftazidime	2734	≤8	≥64	≤1 to ≥64	54.9	10.6	34.5
Imipenem	461	1	16	0.12 to ≥32	66.8	15.0	18.2
Levofloxacin	2734	2	≥16	0.015 to ≥16	52.6	6.1	41.4
Meropenem	2273	2	≥32	≤0.06 to ≥32	64.2	9.6	26.2
Minocycline	2734	16	≥32	≤0.5 to ≥32	--	--	--
Pip/taz	2734	16	≥256	≤0.06 to ≥256	75.3	0.0	24.7
Tigecycline	2734	8	≥32	≤0.008 to ≥32	--	--	--

S, susceptible; I, intermediate; R, resistant; amoxi/clav, amoxicillin-clavulanate; pip/taz, piperacillin-tazobactam.

-- No CLSI breakpoints available.

^a^ The ceftazidime testing range against the Enterobacteriaceae started at 8 μg/mL, therefore susceptible and intermediate classifications can not be calculated.

^b^ The ceftazidime testing range against *H. influenzae* started at 8 μg/mL, therefore a susceptible classification can not be calculated.

^c^ 0.04%, 1 isolate, collected in 2009. The isolate was collected in Mexico in 2009 from a male inpatient. The isolate was also resistant to amoxicillin-clavulanate, ampicillin, ceftriaxone, and minocycline.

**Table 2:** Antimicrobial susceptibility (%S) to the carbapenems among Gram-negative organisms collected from individual countries (2004 – 2010)

		**Country**								
**Antimicrobial**		**Argentina**	**Brazil**	**Chile**	**Colombia**	**Guatemala**	**Honduras**	**Mexico**	**Panama**	**Venezuela**
**non-ESBL *E. coli***										
Imipenem	N	216/219	40/40	47/47	67/71	-	-	64/64	-	21/21
	%S	98.6	100	100	94.4	-	-	100	-	100
Meropenem	N	448/449	164/164	130/130	386/390	174/182	45/46	569/582	84/84	165/165
	%S	99.8	100	100	99.0	95.6	97.8	97.8	100	100
**ESBL *E. coli***										
Imipenem	N	28/29	10/10	29/29	17/18	-	-	51/52	-	-
	%S	96.6	100	100	94.4	-	-	98.1	-	
Meropenem	N	71/72	32/33	65/65	38/40	76/81	30/31	337/346	16/16	27/29
	%S	98.6	97.0	100	95.0	93.8	96.8	97.4	100	93.1
**non-ESBL *K. pneumoniae***										
Imipenem	N	120/121	17/17	29/29	34/35	-	-	47/47	-	10/10
	%S	99.2	100	100	97.1	-	-	100	-	100
Meropenem	N	297/303	88/92	63/67	290/316	83/93	16/20	495/516	53/54	156/163
	%S	98.0	95.7	94.0	91.8	89.2	80.0	95.9	98.1	95.7
**ESBL *K. pneumoniae***										
Imipenem	N	91/93	20/23	35/35	16/18	-	-	19/19	-	-
	%S	97.8	87.0	100	88.9	-	-	100	-	-
Meropenem	N	170/177	78/82	102/112	47/63	76/96	50/55	160/172	32/35	28/31
	%S	96.0	95.1	91.1	74.6	79.2	90.9	93.0	91.4	90.3
***K. oxytoca***										
Imipenem	N	32/32	-	-	13/13	-	-	11/11	-	-
	%S	100	-	-	100	-	-	100	-	-
Meropenem	N	38/38	17/17	15/15	37/38	-	-	106/110	-	-
	%S	100	100	100	97.4	-	-	96.4	-	-
***Enterobacter *spp.**										
Imipenem	N	210/222	44/47	58/58	56/59	-	-	58/58	-	25/25
	%S	94.6	93.6	100	94.9	-	-	100	-	100
Meropenem	N	494/502	187/195	161/171	347/384	83/105	29/34	622/651	66/70	176/183
	%S	98.4	95.9	94.2	90.4	79.0	85.3	95.5	94.3	96.2
***S. marcescens***										
Imipenem	N	83/91	18/20	30/31	37/41	-	-	23/26	-	10/11
	%S	91.2	90.0	96.8	90.2	-	-	88.5	-	90.9
Meropenem	N	203/210	77/78	70/71	138/144	42/45	14/15	220/234	25/25	69/70
	%S	96.7	98.7	98.6	95.8	93.3	93.3	94.0	100	98.6
***A. baumannii***										
Imipenem	N	72/148	13/30	35/39	21/35	-	-	30/30	-	7/11
	%S	48.6	43.3	89.7	60.0	-	-	100	-	63.6
Meropenem	N	48/321	29/118	37/139	95/220	43/141	14/51	202/333	8/48	21/96
	%S	15.0	24.6	27.0	43.2	30.4	27.5	60.7	16.7	21.9

^a^ Data on El Salvador, Jamaica and Nicaragua not included in the analysis by country because fewer than 50 isolates collected.

The most active antimicrobial agents against non-ESBL producing *K. pneumoniae* were tigecycline (MIC_90_ 1 μg/mL), carbapenems (imipenem MIC_90_ 0.5 μg/mL and meropenem MIC_90_ 0.25 μg/mL) and amikacin (MIC_90_ 8 μg/mL) (Table 1). All tested antimicrobial agents displayed reduced activity against ESBL-producing *K. pneumoniae*, with only imipenem and tigecycline recording percentage susceptibilities of >90% (96.0% and 93.7%, respectively). In particular, susceptibilities to levofloxacin against ESBL-producing isolates of *E. coli* and *K. pneumoniae* were lower when compared with non-ESBL-producing strains (11.5% vs. 60.9% and 38.2% vs 80.1%, respectively) (Table 1). Among *K. pneumoniae* 35.3% were ESBL producers and percentages ranged from 17.2% (36/209) in Venezuela to 73.3% (55/75) in Honduras (Figure 1). Both ESBL and non-ESBL-producing *K. pneumoniae* displayed higher resistance levels to carbapenemes than *E. coli* in all countries (Table 2).

Amikacin, carbapenems and tigecycline were the most active agents against *K. oxytoca* (>94% susceptibility) and *Enterobacter* spp. (>89% susceptibility). Against isolates of *S. marcescens* the carbapenems and tigecycline were the most active agents (>91% susceptibility) (Table 1). Among these three species rates of susceptibility to the carbapenems were ≥90% in all countries where data were available, with the exception of susceptibility to meropenem among isolates of *Enterobacter* spp. collected in Guatemala and Honduras (79.0% and 85.3%, respectively) and susceptibility to imipenem among isolates of *S. marcescens* from Mexico (88.5%) (Table 2).

Almost all of antimicrobials in the panel were active against *H. influenzae* with susceptibility varying from 78.7% for ampicillin to 100% for ceftriaxone, imipenem, levofloxacin, and meropenem (Table 1). Almost 20% of isolates (181/908) were β-lactamase producers.

For *A. baumannii* susceptibility was less than 50% for seven of the nine antimicrobial agents (Table 1). The most active agents were minocycline (89.4%, MIC_90_ 8 μg/mL) and imipenem (62.5%, MIC_90_ ≥32 μg/mL). Tigecycline showed good activity against *A. baumannii*: although no breakpoints are available for this agent, 95.8% of the isolates displayed an MIC ≤2 μg/mL. Low rates of carbapenem susceptibility were observed in most countries (Table 2); the lowest rates were reported for meropenem among isolates from Argentina (15.0%) and Panama (16.7%). A total of 600 isolates (33.2%) were multidrug-resistant, among them the MIC_90_ for minocycline and tigecycline were 8 and 2 μg/mL, respectively.

Among *P. aeruginosa* collected the most active agents were piperacillin-tazobactam, with 75.3% of isolates susceptible (MIC_90_ ≥256 μg/mL), and amikacin with 71.8% (MIC_90_ ≥128 μg/mL) (Table 1).

## Discussion

This study reports on rates of antimicrobial susceptibility among important Gram-negative organisms collected from centers in Latin America between 2004 and 2010. It provides an update to the work of Rossi et al. [7] who reported on Gram-negative and Gram-positive organisms collected as part of T.E.S.T. between 2004 and 2007. The isolates reported on by Rossi et al. [7] are also included in the dataset studied in this report. Rates of ESBL-producing *E. coli* and *K. pneumoniae* are similar to the mentioned study and are also similar to those reported by Villegas et al. [3] for Latin American isolates collected in 2008 as part of the SMART study.

This study shows important variations in the rate of ESBL production by country, reaching values around 40% in *E. coli* and >50% for *K. pneumoniae*, which are similar to those observed in the Asia/Pacific region by Farrell et al. [12] for both organisms and by Hawser et al. 2009 [13] for *E. coli*. However, it should be noted that these rates may be affected by the type of infection and population analyzed in each particular center or even by ward [2]. Considering that these are common nosocomial pathogens causing severe morbidity and mortality in critically ill patients and that the available choices of antibiotic treatments for these microorganisms are seriously reduced, there is increasing clinical concern for successful patient management where ESBL isolates are prevalent. Antimicrobial susceptibility rates were lower among ESBL-producing isolates when compared with non-ESBL producers with the exception of tigecycline, imipenem and meropenem where little or no changes in susceptibility (<6.0%) were observed between both groups. ESBL-producing *K. pneumoniae* are frequently associated with multidrug resistance [14]. In particular, susceptibility to commonly-used antimicrobials including piperacillin-tazobactam and fluoroquinolones was reduced among ESBL-producing isolates. The worrying increase in resistance to these antibiotics among ESBL-producing organisms has been associated with the simultaneous presence of other resistance determinants [15-17]. The most common risk factor for resistance to fluoroquinolones in ESBL-producing strains is a previous history of high-level consumption of both extended-spectrum cephalosporin and quinolone antibiotics. These antibiotics are widely used in the region: Wirth et al. reported an increased use of fluoroquinolones in Latin America over a period of 10 years (1997–2007), where in some countries consumption doubled or even tripled [18].

It has been previously reported that tigecycline and carbapenems, along with amikacin, are highly active against the Enterobacteriaceae collected from countries in Latin American [19,20]. In the current study, susceptibility to tigecycline ranged between 99.8% against ESBL-producing *E. coli* to 93.7% against ESBL-producing *K. pneumoniae*. Imipenem susceptibility ranged between 100% against *K. oxytoca* to 91.7% against *S. marcescens* and meropenem susceptibility ranged between 98.6% against non-ESBL-producing *E. coli* to 89.0% against ESBL-producing *K. pneumoniae*. The range of tigecycline MICs was greater than reported by Rossi et al. [7] against *E. coli*, *K. pneumoniae*, and *Enterobacter* spp.; however, this was due to single isolates at the top of the testing range (MIC ≥32 mg/L).

It is worth noting that resistance to meropenem has been observed across Latin America among members of the Enterobacteriaceae. The situation may not appear as poor for imipenem, with higher rates of susceptibility reported. However, it should be noted that imipenem susceptibility testing stopped in 2006 and switched to meropenem, meaning that the results for meropenem give a more current picture of carbapenem susceptibility in Latin America. In the late 1990s and early part of the 21^st^ century, carbapenem resistance in Enterobacteriaceae was infrequent and resistance mechanisms were related to the presence of ESBL or overproduction of AMP-C β-lactamases associated with reduced outer membrane permeability [21,22]. Enterobacteriaceae producing carbapenemases were first reported in the USA [23] and have now been reported in various parts of the world, including several countries in Latin America where class A carbapenemase KPC-2 enzymes are prevalent [5,24-26]. The results of this study, along with reports of decreasing susceptibility to imipenem among *Klebsiella* spp. in Latin America [27] demonstrate the importance of antimicrobial resistance surveillance and further analysis of the carbapenem-resistant Enterobacteriaceae identified in this dataset is warranted.

*H. influenzae* are frequently susceptible to available antimicrobials. In this study susceptibility was >98% to the agents tested, with the exception of ampicillin (78.7% susceptible) largely due to the production of β-lactamase. This is in agreement with the global T.E.S.T. findings published by Garrison et al. [28].

*A. baumannii* is a problematic organism frequently associated with multidrug resistance and 33.2% of the isolates in this study were defined as such. The antimicrobial with the highest rate of susceptibility against the whole *A. baumannii* population was minocycline. Tigecycline was also active, with 95.8% of isolates displaying an MIC ≤2mg/L. These results are similar to those reported by Rossi et al. [7] for Latin America isolates collected between 2004 and 2007 and Garrison et al. [24] who reported on a global collection from the T.E.S.T. study collected between 2004 and 2007. Susceptibility to the carbapenems was 62.5% for imipenem and 33.9% for meropenem which are lower than the global rates reported by Garrison et al. (82.3% and 59.0%, respectively) and lower than the Latin American rates reported by Gales et al. [29] for *Acinetobacter* spp. collected between 2001 and 2004 (86.4% and 83.6%, respectively). Susceptibility also varied by country, Tognim et al. [30] reported as part of the SENTRY study that carbapenem resistance among *Acinetobacter* spp. varied between countries within Latin America with Argentina a particular ‘hot spot’ of resistance. Our results suggest this is a continuing situation with the lowest rates of susceptibility to meropenem reported among isolates from Argentina.

## Conclusions

Surveillance of antimicrobial susceptibility plays a key role in guiding appropriate antimicrobial therapy. In this study the carbapenems and tigecycline continue to be active against the Enterobacteriaceae and *A. baumannii*; however, there is cause for concern with carbapenem non-susceptible isolates reported in all countries included in this study. The in vitro activity (MIC_90_) of tigecycline was similar to that reported for isolates collected during Phase 3 clinical trials [31].

## Competing interests

LFC has received speaking fees from Wyeth Pharmaceuticals (which was acquired by Pfizer Inc. in October 2009). MJD is an employee of Pfizer Inc.

## Authors’ contributions

LFC was involved in data collection, data interpretation and drafting and reviewing of the manuscript; MJD was involved in study design and participated in data interpretation and the drafting and review of the manuscript. All authors read and approved the final manuscript.
